# What’s Happening in the Exam Room? A Mixed-Methods Study About the Provision of Patient-Centered Contraceptive Care for Baltimore Latine Patients

**DOI:** 10.3390/healthcare14111590

**Published:** 2026-06-05

**Authors:** Diana N. Carvajal, Kristin G. Bevilacqua, Christine Dehlendorf

**Affiliations:** 1 Department of Family Medicine and Community Health, School of Medicine and Public Health, University of Wisconsin-Madison, Madison, WI 53705, USA; 2 Department of Population, Family & Reproductive Health, Johns Hopkins Bloomberg School of Public Health, Baltimore, MD 21205, USA; 3 School of Medicine, University of California San Francisco, San Francisco, CA 94143, USA

**Keywords:** contraception, patient-centered care, Latinas, Latine, Hispanic

## Abstract

**Context:** Patient-centered contraceptive care prioritizes patient contraceptive needs and preferences while considering important socio-political factors. Latina/e patients desire such care but experience it inconsistently. This study explores experiences of contraceptive care using quantitative and qualitative methods, including the association between patient characteristics and receipt of patient-centered contraceptive care, among a sample of Latina/e patients in Baltimore, Maryland. **Methods:** We quantitatively assessed experiences of patient-centered contraceptive care among Latina/e patients ages 15–45 by using the Patient-Centered Contraceptive Counseling measure (PCCC) and a visit-satisfaction measure. Audio-recordings of visits were coded using the Four Habits Coding Scheme (4HCS), a measure of patient-centered communication between patients and clinicians. Analyses between patient characteristics and PCCC, 4HCS, and patient satisfaction scores were bivariate and exploratory. Qualitative interviews with a patient subset further characterized experiences. **Results:** A total of 52 visits, including patient surveys, were analyzed; a subset of 29 patients participated in interviews. A majority of patients (63.5%) reported the highest possible PCCC ratings, and 85% reported the highest level of satisfaction with their contraceptive counseling visits. Researcher-reported 4HCS scores were lower than patient-reported measures; scores for one particular habit of the 4HCS were significantly lower for Spanish-preferring and uninsured patients. The discrepancy between patient-reported PCCC and satisfaction ratings and researcher-reported 4HCS scores is contextualized by interviews in which patients reported that counseling received during the study period focused on their contraceptive preferences and involved interactions with friendly, communicative clinicians/staff, which contrasted with previous, lower quality, healthcare experiences. **Conclusions:** Positive experience and satisfaction ratings of Latina/e patients in the context of suboptimal patient-centered care (PCC) on audio-recordings reveal somewhat of a discrepancy between self-report and observation. Provision of contraceptive patient-centered care requires attention to both patient-reported experiences and measures using direct observations, as well as a more robust understanding of how PCC is conceptualized by patients and delivered by clinicians.

## 1. Introduction

Patient-centered care (PCC)—defined as “providing care that is respectful of, and responsive to, individual patient preferences, needs, and values and ensuring that patient values guide all clinical decisions,” is a core component of quality healthcare as defined by the National Academy of Medicine [1]. In the context of contraceptive care, research has demonstrated that PCC must include preference-focused counseling with active, engaged, and comprehensive patient–clinician communication about contraceptive options [2,3,4]. Patient-centered contraceptive care can facilitate patient autonomy, trust in clinicians, and greater satisfaction with care [4,5].

Research has documented that, consistent with the principles of PCC, Latina/e patients desire clear patient–clinician communication focused on their preferences [3]. However, limited research suggests that Latina/e patients do not consistently experience patient-centered contraceptive counseling [3,6,7,8,9]. In the 2017–2019 National Survey of Family Growth, researchers found that Spanish-preferring Latinas reported significantly lower patient-centered care scores compared to those of non-Latina White respondents. Further, only 51% of high-English and 35% of low-English proficiency Latina/e patients reported excellent overall patient-centered contraceptive care [9].

Notably, research also demonstrates that Latinas experience racial/ethnic discrimination in contraceptive services, are more likely than White women to encounter clinician coercion to use contraception and pressure to limit family size and are less likely to experience PCC [10,11,12]. The U.S. has a longstanding legacy of reproductive abuse, including contraceptive coercion that has targeted specific individuals with the capacity for pregnancy including those who are low-income, people of color, and/or immigrants, among others [13,14]. In contemporary times in a study of cis women in San Francisco, 24% of Hispanic/Latinx participants reported experiencing reproductive coercion compared to 18% of White women [15]. Additionally, Latina/x patients are more likely to report LARC coercion and shorter contraceptive counseling visits than their White counterparts [16]. Given these findings, attention to the provision of non-coercive contraceptive decision support is necessary for the delivery of high-quality, equitable care, especially for marginalized populations, including Latina/e, who have been historically denied control of their childbearing [4,13,15]. Yet, observational research on whether and how patient-centered contraceptive care is delivered to Latine patients and how, in turn, Latine patients then perceive their care, remains scarce and elusive.

To address this gap, this study uses multiple measures from both patient and clinician perspectives as means to garner more robust data about how clinicians deliver patient-centered contraceptive care and how patients perceive and receive patient-centered contraceptive care. We therefore measured patient-centered care using a variety of different measurements seeking to capture both the observations of researchers who coded clinician behavior as they delivered contraception counseling as well the perspective of patients as they received care. While, there is no clear existing evidence about how discrepancies between measurements should be interpreted, we were able to describe the extent to which clinician behavior aligned with standard definitions of patient-centered care as well as the degree to which patient’s expectations were met by the care provided and the extent to which clinician behavior aligned with standard definitions of patient-centered care. We then performed qualitative data collection with patient interviews to further explore patient experiences of the interactions and to contextualize quantitative findings.

## 2. Materials and Methods

### 2.1. Recruitment and Data Collection

We recruited Latina/e patients and their clinicians in Baltimore, MD, between January 2022 and January 2024. Recruitment was conducted in two stages. In stage 1 (clinician recruitment), a convenience sample of physicians and nurse practitioners (N = 6) across three specialties (Family Medicine, Pediatrics, and Obstetrics/Gynecology) were recruited from three healthcare facilities that serve predominantly low-income, uninsured patients or those with public insurance. Site 1 is a city health department facility focused on providing reproductive healthcare and serving a predominantly Latine population. Site 2 is an academic pediatric medical practice also with a significant Latine patient population. Site 3 is an academic obstetrics and gynecology practice. Eligible clinicians were recruited via emails sent by facility medical directors or from site-specific clinical staff meetings during which the research team described the study objectives and activities and obtained contact information for interested participants. Clinicians were eligible if they provided contraceptive counseling to Spanish or English-speaking Latine patients. Informed consent was completed via Research Electronic Data Capture (REDCap) v14.5, a software platform for clinical research that is commonly used to create and manage questionnaires [17]. Clinicians were asked to complete a baseline survey, and each received an $85 gift card as remuneration.

For stage 2 (patient recruitment), recruitment varied by site. For sites 2 and 3, the study research assistant (RA) reviewed clinicians’ weekly schedules via the electronic medical record to identify eligible patients and then recruited patients who clinicians acknowledged were appropriate for the study, recognizing the potential for selection bias and encouraging clinicians to consider all/most patients as appropriate if eligibility criteria was met. For recruitment at site 1, an office administrator sent a weekly email to the RA with a de-identified list of appointments, which included the date, time, and reason for the appointment.

Patients were eligible for study participation if they were ages 15 to 45 years, identified as Hispanic/Latina/e/x, spoke English or Spanish, and were not actively trying to become pregnant. Once assented or consented to participate in the study, patients completed a pre-visit survey. Their contraceptive counseling visits were then audio-recorded, and patient participants completed a post-visit survey immediately following the visit at each clinic site. Each patient participant received a $60 gift card as remuneration.

To provide additional and more nuanced insight into patient experiences, we conducted qualitative interviews with a subset of patients who agreed to be contacted for follow-up to discuss their visit experiences. All patients were invited to participate in an interview if desired. Those who expressed interest and agreed to participate in interviews were contacted via phone to schedule an interview date. In-depth interviews were conducted with the subset of patient participants who consented (N = 29) via telephone using a semi-structured interview guide informed by the main tenets of patient-centered care [1]. Interviews were conducted by the study research assistant (second author, K.B.), a White, non-Latina cisgender woman who speaks Spanish as a second language. The interviews were recorded using a study-dedicated Google Voice account; each patient who agreed to participate in an interview received an additional $40 gift card.

### 2.2. Outcome Measures

#### 2.2.1. Patient-Centered Contraceptive Counseling

Patient-centered counseling was measured using patient-reported measures and by the coding of audio-recordings. These two approaches were used to capture subjective patient experiences as well as direct observation of the quality of care. Patient reports and observation have been found to produce weakly correlated results, indicating they capture different dimensions of communication that are both of interest. Patient experience of care is inherently a valuable outcome, as it assesses patients’ own assessment of whether their needs were met, while observed behaviors provide more objective information about the care being provided [18]. These approaches provide complementary but distinct perspectives [19]. First, patient post-visit surveys included the 4-item Patient-Centered Contraceptive Counseling Scale (PCCC) [20]. Consistent with standard use of this measure, scores were dichotomized using either a (1) rating of excellent for all four items or (0) less than excellent for any of the four items [20]. Importantly, in previous research, the desired favorable threshold for PCCC ratings is that at least 80% of respondents select excellent for all four items [20,21].

We also assessed patient satisfaction using a single item from the Patient–Provider Communication about Contraception study [21] to capture a holistic evaluation of patient expectations and opinions of the care received. This item asked participants to rate their satisfaction with the contraceptive counseling they received: “Overall, how satisfied are you with how your health care provider helped you choose the birth control method you would use?”. They used a 7-point Likert response scale ranging from “completely unsatisfied” (0) to “completely satisfied” (7) or “I did not choose a method today” (8). Due to very positively skewed responses, the patient satisfaction variable was collapsed to (1) completely satisfied and (0) not completely satisfied (any rating less than 7).

Second, patient-centered counseling was quantitatively assessed by coding audio-recordings of contraceptive care visits. Verbatim transcripts were independently reviewed by two study team members and coded using the Four Habits Coding Scheme (4HCS). All visits were dual-coded shortly after they were collected, and study team members met weekly to compare coding for each individual visit. Discrepancies were discussed, and consensus was met to produce final item-level 4HCS scores. Because of this iterative, collaborative process, no inter-relater reliability scores were calculated.

The 4HCS is a 23-item inventory of patient-centered clinician behaviors and skills and is associated with effective clinical practice and positive health outcomes [21]. We used a previously adapted version of the 4HCS to assess patient-centered contraceptive counseling [22]. Contraceptive counseling-adapted Habits 1 (“Invest in the beginning”), 2 (“Elicit the patient’s perspective”), and 4 (“Invest in the end”) were used; however, Habit 3 (“Demonstrate empathy”) was not included in the analysis, as the items were inconsistently applicable to the specific clinical encounters observed. For example, this habit stipulates that clinicians should “encourage appropriate expression of emotion” and “show empathy for patient’s experiences or feelings.” However, most audio-recordings did not convey significant patient emotions or feelings in which one would reasonably expect a clinician to respond with or display empathy. Although, we refer to the measure as the Four Habits Coding Scheme (4HCS) throughout the manuscript, we recognize this study’s partial adaptation of the instrument; simultaneously, we assert the necessity of the adaptation given that application of coding for the Habit (3) was simply inappropriate and of little use in most instances. Coding options for each item range from 1 “highly effective” to 5 “not effective.”

Highly effective counseling sessions for Habit 1 (“Invest in the beginning”) included those in which the clinician exhibited a warm/friendly tone, engaged in brief non-medical conversation, used open-ended questions throughout the visit, and encouraged discussion about the patient’s medical concerns. Highly effective counseling sessions for Habit 2 (“Elicit the patient’s perspective”) were those in which the clinician extensively explored a patient’s past experiences with contraception as well as their current contraceptive preferences using open-ended questions—even when the patient did not initially bring them up. Finally, for Habit 4 (“Invest in the end”), highly effective sessions were those in which the clinician intentionally and actively ensured that patients absorbed the information being provided, encouraged them to ask questions, and checked for understanding while not speaking too fast and/or interrupting [22]. Scores for each habit of the 4HCS were dichotomized based on previous research as (0) less effective and (1) highly effective, with a highly effective score indicating visits scoring in the top quartile of effectiveness [21]. Fifty-two visits among five clinicians across three sites were independently coded by two study team researchers following the 4HCS rubric [21] after listening to the audio-recording of each visit; these two researchers then met on a weekly basis to discuss coding decisions and resolve conflicts.

#### 2.2.2. Independent Study Variables

For clinician participants, demographic and medical specialty and training data were collected in the baseline survey. For patient participants, the pre-visit survey collected demographic information, including age, gender, race, country of origin, parents’ country of origin, year of immigration to the United States, educational attainment, employment status, relationship status, and whether the patient had a prior visit to the facility.

### 2.3. Analysis

#### 2.3.1. Quantitative Analysis

Descriptive statistics were recorded for both clinician and patient demographic characteristics. T-tests were used for continuous variables and chi2 was used for categorical variables to assess associations of patient demographic characteristics with patient satisfaction, PCCC scores, and 4HCS scores.

Multivariable fixed effects logistic regression was conducted to examine the association between contraceptive counseling rating scores and location, language preference, and insurance status. Due to the small number of clusters (clinicians) and limited within-cluster sample sizes (patients), fixed-effects models were preferred over random-effects specifications to minimize potential bias from unobserved cluster-level heterogeneity and to avoid reliance on assumptions that may not hold in small samples. STATA 18 was used to conduct analyses.

#### 2.3.2. Qualitative Analysis

Interviews were audio-recorded, transcribed verbatim, and analyzed using a directed contact analysis approach [23] based on the main tenets of PCC. Following the constant comparison method [24], two study team members independently reviewed the first 10 transcripts and developed an initial hybrid inductive/deductive codebook focusing on participant experiences of PCC, including reports of positive communication, adequate information exchange with clinicians, a focus on preferences, friendly/kind interactions, feelings of trust, and non-coercive decision-making. All team members have extensive training and experience with qualitative studies and met regularly to revise the codebook as new interviews were conducted to explore theoretical connections between existing and new interviews, discuss deviant cases, and update the codebook with emerging themes. Coding conflicts were discussed and resolved by eventual consensus, allowing for evolution and refinement of the coding scheme. Data collection concluded once data saturation was reached; subsequently, any remaining interviews were completed, and the codebook was finalized and applied to the remaining transcripts by one study team researcher. Throughout data collection and analysis, the RA (K.B., second author) and the first author met regularly to discuss how K.B.’s positionality influenced her interviewing and analysis, with careful attention to power dynamics, cultural differences, and the need to center participants’ voices. All coding was conducted using Atlas.ti (Headquarters: Berlin, Germany) Desktop for Macbook (V 24.1.1) [25]. Following coding completion, data were reviewed and organized around emergent themes, presented below.

## 3. Results

### 3.1. Quantitative Results

Six clinicians across three sites completed at least one recorded contraceptive counseling visit with an enrolled patient. Fifty percent of clinicians were 30–40 years old, 50% completed their training within the prior 11–19 years (Table 1), and two-thirds were MD/Dos. Most clinicians (83%) identified as White, 17% as Black/African American, and 17% as Latina/x (non-mutually exclusive categories). Half of clinicians indicated that they speak Spanish “somewhat.”.

A total of 109 patients were approached to participate in the study, and 61 (56%) consented to participate. Of the 61 patients, nine did not receive contraceptive counseling of any kind, while 52 did receive counseling. Among those who received counseling, 41 chose a contraceptive method during their visit. Most patients were 18 years of age or older (85%), preferred Spanish (67%), and were born outside of the US (73%). Seventy-one percent had a high school education or higher at the time of data collection, and 58% were employed or in school. Additionally, most (67%) were uninsured at the time of study participation. See Table 2.

**Figure 1:**
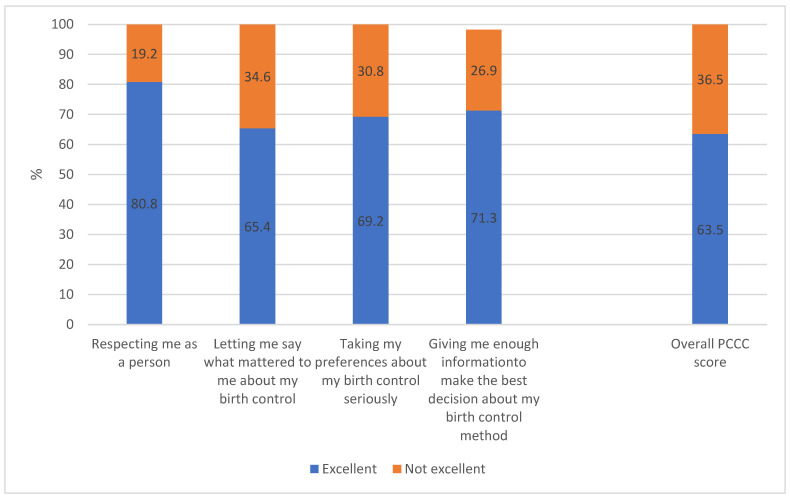
Patient-Centered Contraceptive Counseling ratings by item and overall score (N = 52). Overall PCCC score: all items scored excellent versus any items not scored as excellent.

**Figure 2:**
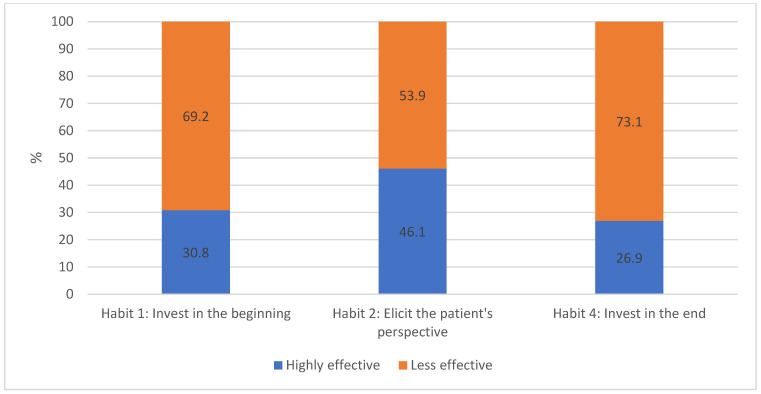
Four Habit Coding Scheme ratings by item (N = 52).

Patient-reported PCCC scores were skewed toward positive ratings, with no patient endorsing poor or fair for any items and excellent scores for the four items ranging from 65% to 81% of participants (Figure 1). Overall, 64% of all participants scored their clinician as excellent on all four items of the PCCC. Patient satisfaction with how the clinician helped them choose their contraceptive method was highly skewed toward complete satisfaction for those who chose a method (N = 41), with 85.4% (N = 35) completely satisfied. For the Four Habits Coding Scheme, 31% of counseling sessions were coded highly effective for Habit 1, 46% for Habit 2, and 27% for Habit 4 (Figure 2).

In bivariate analyses (Table 2), top box PCCC scores (all excellent) and highly effective Habit 2 and Habit 4 scores did not vary significantly by any patient characteristic. For Habit 1, highly effective scores were significantly more likely among English-speaking patients and insured patients. Patient satisfaction with contraceptive counseling was not associated with any demographic characteristic.

In multivariable analyses, clinician location, patient language preference, and insurance status were not associated with contraceptive counseling rating scores (see Table 3). Because clinician location and insurance status perfectly predicted the Habit 2 scores, these categories were omitted from the model, and the corresponding observations were excluded due to complete separation.

### 3.2. Qualitative Results

Three key themes were identified to contextualize patient ratings of their contraceptive care experience: (1) patient satisfaction with counseling reflects a preference for care aligned with patient-centered care principles; (2) prior negative experiences of medical care shapes current counseling expectations, experiences, and satisfaction among patient participants; and (3) well-established patient–clinician relationships foster trust and satisfaction with contraceptive counseling.

**Theme 1: Patients’ counseling preferences aligned with patient-centered counseling**

While no participant specifically identified a preference for “patient-centered contraceptive counseling” by name, reasons for high satisfaction with counseling closely align with the major tenets of patient-centered care, namely high-quality communication and respect for patient values and preferences [26].

Nearly every study participant described the friendliness and kindness of clinicians and staff as contributing factors to their high satisfaction with the visit:

[…] The impression I got is that I received good service. The nurse, doctors [were] very kind. They were very kind people. (Site 1, translated from Spanish)

Positive interpersonal interactions (which included kindness), in turn, engendered trust and comfort from the point of view of participants:

[…] the way in which she [clinician] spoke to me, she was very kind. And because of that, I think [I am] very comfortable with her. (Site 3, translated from Spanish)

There was zero judgment at all from her. Didn’t make me feel like it was odd or weird or something I shouldn’t even be thinking about. So, it was like very easy to be open and ask her what I was looking for. (Site 2)

Participants expressed the interconnection between quality communication and positive experience of the visit:

They gave me the information I wanted, that I asked for. She explained things to me the best she could, enough for me to feel satisfied with what she was telling me. And well, for me, I felt good during the visit. (Site 1, translated from Spanish)

Relatedly, participants were satisfied with the availability of counseling in their preferred language, either via interpreter services or directly from the clinician. Notably, even when interacting with clinicians who have limited Spanish proficiency, participants expressed appreciation for clinicians’ efforts to communicate in Spanish and felt that they were still able to achieve mutual understanding:

[…] In terms of communication, she [clinician] spoke to me more in English than in Spanish. So, when she saw that she couldn’t say something in Spanish, she would say it to me in English. I understand her a bit, and either way, she found a way that we could understand one another. (Site 1, translated from Spanish)

Lastly, participants described clinician support and respect for their method choice; participants were satisfied with their access to those methods:

I feel great because [preferred method] was already on my mind and then talking about all of the birth control options and all the differences she gave for each one, I felt like I just gravitated towards that one more, and she was definitely in support of it too. […] It was nice to collectively come to that decision and have the information that I needed to fully commit to that one. (Site 2)

**Theme 2: Prior healthcare experiences shape counseling expectations and experiences**

Several participants described previous negative experiences accessing both contraceptive services specifically and, more broadly, healthcare in general. Some expressed beliefs that these prior negative interactions were shaped by their Latine identities:

I feel within the Latinx community, doctors are like, I’ve actually heard friends talk about this. They’re [clinicians] just a lot more like judgmental about it, like, because pregnancies within our communities are pretty common, especially at younger ages. So, they’re already going into the appointments with that mindset, and it ends up being not easy for people of, like, Hispanic communities to ask the doctors about it […], but I do know it’s a huge issue, and I know people that struggle with that. (Site 2)

In particular, participants described discrimination and mistreatment based on language preferences:

As a Latina that speaks English, like fluent English, I will go with my mom, and they [healthcare staff] would kind of be a little disrespectful because they think they have to force themselves to speak Spanish. […] When I was younger, I would be the person to translate for her. They would treat my mom like, you know like we don’t even want to test you because you’re Latina because you speak Spanish, and that would be something that really made me mad because I was young, and I would see Latinas or Latinos struggle with doctors because they needed to explain themselves. So, as I was growing up, I forced myself to perfectly talk English and have the right vocabulary because it’s really messed up that people would treat Latinos different just because of their language or their accent. (Site 1)

In contrasting their recent counseling visits with previous negative healthcare experiences, notable differences aligned with key tenets of patient-centered care, including respect for patient values and information exchange:

Truthfully, the other times, at other places that I went to have this placed, the birth control—they were not very friendly, honestly. She was very kind, and the others were not so kind. They did not explain as much. They didn’t tell me how it would be. (Site 1, translated from Spanish)

**Theme 3: Well-established relationships foster trust and satisfaction**

Participants expressed that their established relationships with their clinicians and with the staff at trusted healthcare facilities made them feel comfortable discussing sensitive topics—including contraception—and confident in the information and guidance they were given:

[…] Well, since I had been here before, I feel trustful. I feel safe. I feel good. (Site 1, translated from Spanish)

Every time I go [to that clinic], I feel good. They always give me the treatment I want. They gave me my injection. They treat me well. They don’t discriminate against me, and that is all. (Site 1, translated from Spanish).

## 4. Discussion

Results of this study highlight the importance of integrating and accounting for both distinct quantitative measures of PCC as well as qualitative data when considering how patient-centered contraceptive care is delivered by clinicians and experienced by patients. Quantitative analyses of Latine patients’ contraceptive counseling experiences found that overall patient-reported PCCC scores were below the desired threshold [21], while patient satisfaction scores were overall higher, yet evaluation of audio-recordings using the 4CHS identified gaps in the quality of patient-centered contraceptive care. We did not demonstrate any clear associations between patient demographic characteristics and self-reported PCCC and satisfaction scores. However, patient language preference (Spanish) and lack of insurance were associated with researcher-reported less effective 4HCS ratings for Habit 1, “Investing in the Beginning”. PCCC and satisfaction scores overall were generally positive. Qualitative interviews provided important context for these findings, highlighting the value of quality communication focused on patient preferences and continuity of care but also revealing that prior experiences likely affect current patient healthcare experiences.

While extant research directly comparing patient-reported PCCC scores and research-reported 4HCS ratings are limited or non-existent, our findings are consistent with a previous study that found discrepancies when evaluating contraceptive counseling using both patient-reported measures and researcher-reported ratings [27]. As previously noted, limited research has demonstrated that Spanish-preferring Latinas have reported lower self-reported PCCC scores than non-Latina White respondents [8,9]. Moreover, for patients in general, there is likely a social desirability bias in which they tend to rate their clinicians highly, and that potentially skews ratings in a more positive direction compared to researchers.

Yet qualitative results also provide potential explanations for the differences in patient- and researcher-reported ratings. In qualitative interviews, patient participants reported that their current contraceptive counseling experiences were positive in comparison to previous negative healthcare experiences, including prior experiences of discriminatory treatment in healthcare settings. Poor treatment by healthcare staff and clinicians, including experiences of racial/ethnic discrimination, are well documented within the U.S. Latine population [27,28]. Latine individuals born outside of the U.S. and those with limited English proficiency may have even worse experiences [29,30,31]. Extant data also suggests that Latina/e individuals, especially those who are young, experience pressure from clinicians to use methods of contraception that they do not want to use [30]. When describing their contraceptive counseling experiences during our study, participants often reported being treated with respect and that staff and clinicians were friendly and often spoke their preferred language. In addition, several participants reported being given a range of options and did not feel they were forced to use methods they did not want to use. This contrasts with previous healthcare experiences which may, in part, explain the mostly positive ratings across the four items of the PCCC [11,30,32,33]. In addition, given some patients’ previous negative healthcare experiences, an interpretative hypothesis of the study findings could be that current expectations of respectful, patient-centered treatment may be low at baseline [34].

As stated previously, studies using the PCCC have in fact found significant differences by patient demographics, including preferred language and income, with lower income and Spanish-preferring patients having lower scores compared to English-preferring patients [8,9,35,36]. Our lack of findings may be related to our small sample size, as there are substantial differences in the point estimates for education, insurance, and language in the expected direction. The lower scores for Spanish-preferring patients for Habit 1, “investing in the beginning”, however, are consistent with prior literature about lower patient-reported PCCC scores among Spanish-preferring patients and those without insurance [35,36], suggesting lower-quality communication for those with less English proficiency.

Additionally, the finding of higher scores on Habit 1 for insured patients may be interpreted in the context of the qualitative results potentially indicating improved experiences of care for patient participants with established clinician relationships. Uninsured patient participants were significantly less likely to have seen their clinician prior to the contraceptive counseling visit (*p* < 0.001). Further, there was a trend towards more positive Habit 1 scores among those with established relationships. This could suggest that one-time or short-term patient–clinician relationships—which were more likely among uninsured patients—may limit a clinician’s opportunity to convey investment in the patient visit and engender trust in a manner that aligns with patient expectations for patient-centered contraceptive counseling. This finding is consistent with extant literature demonstrating the importance of continuity relationships with respect to overall patient satisfaction [37,38,39,40,41]. Unfortunately, continuity of care between patients and clinicians was not feasible for all study participants. For example, a portion of study patients received postpartum contraceptive counseling from a clinician who they had not seen during pregnancy and whom they would no longer be able to see following termination of their pregnancy-related public assistance.

Results must be understood in the context of the study’s limitations. Patient participants were recruited through convenience methods and are not representative of all Latina/e individuals seeking contraceptive care. The same is true of clinician participants. Both patient and clinician participants were recruited in Baltimore City, and their experiences accessing and providing reproductive healthcare, respectively, may not be generalizable outside of this setting. Additionally, for both patient and clinician participants, those who chose to enroll in the study may be systematically different from those who did not—in ways related to their contraceptive counseling experiences and provision. Selection bias among participating clinicians and their patients may have also contributed to the differences in patient-reported and research-reported scores. For example, clinicians may have selected for study participation those patients whom they thought would rate their counseling experiences most favorably (selection bias). Also, of consideration is whether Spanish-preferring participants received the benefit of *Communication and Language Assistance*, which requires providing timely language services for individuals with limited English proficiency [42]. While the authors believe all participants in need of such services were offered them, it was neither measured nor confirmed. Finally, the small sample size contributed to limited power and wide confidence intervals, and importantly, there is limited/no evidence to our knowledge about how discrepancies between patient-reported PCC (via the PCCC) and researcher-observed PCC (via the 4HCS) should be interpreted conceptually. While we offer potential explanations based on available data about what factors could potentially be driving discrepancies, there is no existing standard for directly comparing such data. Nonetheless, multiple sources of data contribute significantly to the richness and robustness of study findings and highlight the importance of multiple forms of data when evaluating both how care is delivered by clinicians and experienced by patients. Multiple data sources provide deeper insight and perspective into the experiences of patients and start to help us tell the full story of how patient-centered contraceptive care is received, perceived, and delivered.

## 5. Conclusions

Ultimately, the evolving trajectory for understanding how patient-centered contraceptive care is experienced by Latina/e patients and how it is delivered by clinicians benefits from the use of qualitative and quantitative approaches. There is clear room for improvement in care delivery, as the PCCC rating fell short of target goals and 4HCS scores indicated gaps in PCC [20,21]. Importantly, the mostly positive satisfaction and PCCC scores may be related to previous poor healthcare experiences and possibly low expectations among participants—which, if true, could mean that the current bar for high-quality care for this population may be too low. It is thus possible but not certain that measures of patient experience with care may not reflect receipt of true patient-centered counseling, especially for communities historically excluded from equitable delivery of care and should be interpreted with caution.

The considerably unfavorable 4HCS ratings for Habits 1 and 4 [invest in the beginning and the end, respectively] suggest that Baltimore clinicians could consider a focus on working intentionally to optimize their delivery of patient-centered contraceptive care for their Latina/e patients by improving initial communication to include primarily open-ended questions and encouraging patients to share their concerns. Extant literature about clinician perspectives demonstrates that they recognize the importance of PCC in contraceptive care [43] and consider elicitation of patient preferences, evidence-based information exchange, and clear communication to be critical components of PCC [43,44]. However, research also demonstrates that clinicians are not always able to provide PCC due to both system-level barriers, such as visit time constraints and racial/ethnic discrimination within the health system, and clinician-level barriers, including patient–clinician language discordance and lack of clinician cultural humility [43,44].

These obstacles can interfere with the application and implementation of PCC in practice. Therefore, there must be intentional approaches to support clinicians to have the training and time to focus on, remind themselves of, and uplift PCC tenets during contraceptive counseling visits, including allowing patients to absorb essential information, encourage additional questions, and include a clear follow-up plan. For Latina/e patients seeking contraceptive counseling, future intervention and research can focus on more robust training for clinicians to establish rapport and practice patient-centered care regardless of a patient’s preferred language or insurance status. The research will pursue their planned next steps for future intervention, which include the development of a clinician-facing point-of-care tool that will strive to guide and facilitate the use of patient-centered care during contraceptive counseling. As we continue our efforts to improve the delivery of patient-centered contraceptive care, particularly for those communities currently least likely to experience it, the results of this study underscore the importance of including and accounting for multiple approaches to investigating patient-centered care for greater understanding of how care is both experienced and delivered.

## Figures and Tables

**Table 1:** Clinician demographic characteristics (N = 6).

Demographic Characteristic	N = 6
**Age**	**col % (n)**
<30 years old	33 (2)
30–40 years old	50 (3)
51–65 years old	17 (1)
**Years since completed training**	
<7 years	17 (1)
7–10 years	17 (1)
11–19 years	50 (3)
≥20 years	17 (1)
**Training**	
MD/DO	67 (4)
Nurse Practitioner	33 (2)
**Site**	
Site 1	17 (1)
Site 2	50 (3)
Site 3	33 (2)
Race/ethnicity *	
Black/African American	17 (1)
Latino/a/x	17 (1)
White	83 (5)
**Gender**	
Cis female	83 (5)
Cis male	17 (1)
**Spanish speaking level**	
Not at all	17 (1)
Somewhat	50 (3)
Very well	33 (2)

* Sum > 100% as clinicians could select more than one race/ethnicity.

**Table 2:** Patient demographics overall and by patient satisfaction and patient-centered contraceptive counseling scores.

	Total	Completely Satisfied	*p*-Value	PCCC Top Box Score	*p*-Value	Habit 1 Highly Effective Score	*p*-Value	Habit 2 Highly Effective Score	*p*-Value	Habit 4 Highly Effective Score	*p*-Value
	N = 52	N = 35		N = 33		N = 16		N = 24		N = 14	
	col %	row % (n)									
**Age, median (IQR)**			0.16		0.63		0.48		0.48		0.81
Less than 18 years	19 (10)	67 (4)		70 (7)		40 (4)		40 (8)		30 (3)	
18 years or older	81 (42)	89 (31)		62 (26)		29 (12)		50 (16)		26 (11)	
**Marital status**			0.23		0.18		0.26		0.27		0.70
Married/committed relationship	67 (35)	90 (26)		57 (20)		26 (9)		51 (18)		29 (10)	
Separated/divorced, casual dating, no relationship	32 (17)	75 (9)		76 (13)		41 (7)		35 (6)		25 (4)	
**Number of children**, median (IQR)	1 (2)	2 (4)	0.77	1 (3)	0.24	1 (3)	0.44	1 (4)	0.97	1 (2)	0.39
**Educational attainment**			0.63		0.11		0.28		0.28		0.16
HS or more	71 (37)	84 (26)		70 (26)		32 (13)		51 (9)		32 (12)	
Less than HS	29 (15)	90 (9)		47 (7)		20 (3)		33 (5)		13 (2)	
**Employment status**			0.17		0.57		0.23		0.60		0.19
Full-time or self-employed	17 (9)	100 (9)		56 (5)		33 (3)		44 (5)		11 (1)	
Part-time	23 (12)	100 (8)		50 (6)		33 (3)		42 (5)		42 (5)	
Student	17 (9)	71 (5)		67 (6)		56 (5)		67 (6)		44 (4)	
Not employed	42 (22)	76 (13)		73 (16)		18 (4)		41 (9)		18 (4)	
**Language preference**			0.70		0.18		0.02 *		0.93		0.34
English	33 (17)	82 (9)		77 (13)		53 (9)		47 (8)		35 (6)	
Spanish	67 (35)	87 (26)		57 (20)		20 (7)		46 (16)		23 (8)	
**Country of birth**			0.47		0.47		0.07 **^f^**		0.77		0.39
U.S.	27 (14)	78 (7)		71 (10)		50 (7)		43 (6)		36 (5)	
Outside U.S.	73 (38)	88 (28)		61 (23)		24 (9)		47 (18)		24 (9)	
**Insurance status**			0.70		0.46		0.00 **		0.93		0.34
Insurance	33 (17)	82 (9)		71 (12)		58 (10)		47 (8)		35 (6)	
No insurance	67 (35)	87 (26)		60 (21)		17 (6)		46 (16)		23 (8)	
**New patient status**			0.39		0.68		0.08		0.48		0.30
Existing	39 (20)	92 (12)		60 (12)		45 (9)		40 (8)		35 (7)	
New	61 (32)	82 (23)		6 (21)		22 (7)		50 (16)		22 (7)	

Ns for patient satisfaction; PCCC and habits include only participants who endorsed the highest category for the respective outcome. Total N for patient satisfaction = 41 participants who chose a method; total N for PCCC and 4 habit scores = 52 participants who received contraceptive counseling. ^f^ *p* < 0.1; * *p* < 0.05; ** *p* < 0.01.

**Table 3:** Multivariable logistic regression of contraceptive counseling rating scores.

	PCCC	Habit 1	Habit 2	Habit 4
	aOR (95% CI)			
**Location**				
Family planning clinic	<ref>			
Pediatrics	0.90 (0.09, 8.54)	0.22 (0.01, 3.79)	omitted	2.31 (0.22, 24.02)
Ob/GYN	0.70 (0.15, 3.31)	0.41 (0.04, 4.36)	omitted	3.77 (0.71, 19.96)
**Language preference**				
English	<ref>			
Spanish	0.19 (0.02, 2.53)	1.03 (0.09, 12.24)	0.65 (0.05, 7.89)	0.71 (0.06, 8.50)
**Insurance status**				
Insurance	<ref>			
No insurance	2.29 (0.15, 35.56)	0.05 (0.00, 1.13)	omitted	1.14 (0.06, 21.31)

## Data Availability

The data presented in this study are available on request from the corresponding author. The data are not publicly available due to privacy or ethical restrictions.
